# Molecular and Physiological Insights into CAT- and SOD-Associated Redox Homeostasis Under Salt Stress in *Artemisia argyi*

**DOI:** 10.3390/ijms27177748

**Published:** 2026-08-29

**Authors:** Airish Nayab, Atif Ayub, Fatima Urooj, Ayesha Ayub, Kamran Ahmad, Zhao Yipeng, Sabbir Ahmed, Hamza Sohail, Yongjun Wu

**Affiliations:** 1 College of Life Sciences, Northwest A&F University, Yangling 712100, China; 2 College of Agronomy, Northwest A&F University, Yangling 712100, China; 3 Department of Bioinformatics and Biotechnology, Government College University Faisalabad, Faisalabad 38000, Pakistan; 4 College of Horticulture and Forestry, Tarim University, Aral 843300, China; 5 Xinjiang Production & Construction Corps Key Laboratory of Facility Agriculture, Tarim University, Aral 843300, China

**Keywords:** antioxidants, osmoprotection, photosynthesis, ROS, synteny, transcriptomics

## Abstract

Soil salinity disrupts redox homeostasis and limits plant growth and development. Although catalase (CAT) and superoxide dismutase (SOD) are key enzymatic antioxidants, the *CAT* and *SOD* gene families have not been characterized in *Artemisia argyi* (*A. argyi*), a species of medicinal and ecological importance. While SOD and CAT serve as the primary enzymatic scavengers for reactive oxygen species (ROS) detoxification, their genomic architecture and stress-responsive regulatory networks in *A. argyi* have remained uncharacterized. In this study, we conducted the first comprehensive genome-wide analysis of these gene families in *A. argyi*, identifying 22 structurally conserved members (8 *AarCATs* and 14 *AarSODs*). Collinearity and synteny analyses revealed strict lineage-specific evolutionary conservation, while tertiary protein modeling and subcellular localization illustrated a highly organized multi-organelle defense compartmentalization. High salinity (up to 200 mM NaCl) reduced the stomatal conductance and net photosynthetic rate. Salt stress reduced growth and increased osmoprotectant and antioxidant accumulation in *A. argyi*. Furthermore, histochemical staining using nitroblue tetrazolium (NBT) and 3,3′-Diaminobenzidine (DAB) provided comprehensive evidence of significant accumulation of ROS in leaves, which indicates the intense oxidative stress triggered by ionic stress. Tissue-specific analysis revealed that *AarCAT1*, *AarCSD1*, and *AarFSD2* were 3.9-, 7.9-, and 12.7-fold higher in leaves than in roots, respectively. Under stress, *AarCAT6* and *AarCSD1* were strongly repressed in leaves by ~50% and ~46–70%, respectively, whereas *AarMSD2* and *AarMSD3* were significantly induced in roots by ~2.2- and ~1.8-fold. These distinct expression patterns suggest their potential involvement in tissue-specific stress adaptation and ROS homeostasis. These findings uncover the evolutionary and physiological basis of salt tolerance in *A. argyi*, providing genetic targets for climate-resilient breeding.

## 1. Introduction

Abiotic stresses, including drought, salinity, and heat, severely constrain plant growth and development [1]. In response to these adverse conditions, plants accumulate ROS, which serve as both critical signaling molecules and potent inducers of oxidative stress when homeostasis is disrupted. ROS, which consist of a superoxide anion, hydrogen peroxide, hydroxyl radical, and singlet oxygen, are produced as a lethal derivative of regular oxygen metabolism and serve as a signaling component in plant molecular biology [2,3]. The ROS-metabolizing enzymes, including SOD, CAT, peroxidase (POD), glutathione peroxidase, ascorbate peroxidase, glutathione reductase, and glutathione S-transferase enzymes, are crucial for ROS depletion during stress. Therefore, maintaining ROS at an optimal homeostatic threshold is crucial for proper cellular signaling, a balance achieved through the equilibrium between ROS production and scavenging [4,5].

To regulate oxidative stress and maintain ROS at a non-toxic level, eukaryotic cells produce a highly coordinated enzymatic defense system comprising enzymes such as SOD, glutathione peroxidase, and CAT [6,7]. Together, SOD, CAT, and POD function as an essential enzyme protection system that blocks the chain reaction of free radicals. Within this enzymatic regulation, SOD and CAT operate in tandem to form the primary biological axis of ROS detoxification. The SOD enzyme has the vital role of being the crucial first line of defense in the antioxidant system [4,8]. SOD catalyzes the initial dismutation of highly reactive superoxide radicals into H_2_O_2_ and molecular oxygen. Because the resulting H_2_O_2_ remains toxic at elevated concentrations, it must be rapidly regulated inside the cell [9].

However, the *SOD* gene family has been extensively identified and characterized in many plants, such as *Eleusine coracana* [10], *Brassica campestris* [9], *Oryza sativa* [11], *Brassica napus* [12], *Brassica juncea* and *B. rapa* [13], *Arabidopsis thaliana* [14], Populus [15], *Solanum lycopersicum* [16], and so on. In medicinal plants, *SOD* genes exhibit stress-responsive expression, and their promoters are significantly enriched with light- and hormone-responsive cis-elements, suggesting their involvement in both environmental signaling and antioxidant regulation. The *SOD* multigene family exists differentially across the cytoplasm, chloroplasts, and mitochondria, enabling a spatially coordinated scavenging network against reactive oxygen species [17,18].

The *CAT* gene family serves as a high-capacity downstream scavenger, utilizing a unique advantage among PODs to specifically and efficiently decompose H_2_O_2_ into harmless water (H_2_O) and oxygen (O_2_) [6,19]. Unlike other peroxide-metabolizing enzymes, CAT catalyzes this disproportionation reaction without the need for a cellular reductant, ensuring the rapid removal of H_2_O_2_ produced during photorespiration, mitochondrial electron transport, and fatty acid β-oxidation. The tetrameric hem-comprising CAT enzyme quickly decomposes H_2_O_2_, producing water (H_2_O) and oxygen (O_2_), and exists in almost all aerobic organisms [20,21]. The activities of *CAT gene family* have been reported in peroxisomes, mitochondria, cytosol, and chloroplasts [4,6,22,23]. In plants, the *CAT* gene family has been characterized and investigated in eggplant (*Solanum melongena*) [24], tomato (*Lycopersicon esculentum*) [25], sweet potato (*Ipomoea batatas* L.) [26], barley (*Hordeum vulgare* L.) [27], tobacco (*Nicotiana tabacum*) [5], maize (*Zea mays* L.) [28], wheat (*Triticum aestivum* L.) [29], rice (*Oryza sativa* L.) [30,31], cucumber (*Cucumis sativus* L.) [32] and cotton (*Gossypium hirsutum* L.) [1].

The *CAT* gene family mitigates abiotic stress by scavenging H_2_O_2_. Beyond detoxification, it sustains ROS homeostasis in light and dark conditions and responds to drought and cold, functions intimately tied to photorespiration. Hence, the expression levels of the *CAT* genes vary in different plant tissues. In hot pepper (*Capsicum annuum* L.), *CAT1* and *CAT3* expression in the leaf and stem are linked to circadian rhythms and different stresses [33]. In cucumber, *CAT1* and *CAT3* expression in different organs, including the root, stem, leaves, flowers, and fruits, is significantly induced by salinity, cold, drought, and ABA treatments [32].

*A. argyi* is a perennial herbaceous plant belonging to the *Asteraceae* family, which comprises over three hundred species widely distributed across Asian countries, including China, Korea, and Japan [34,35]. Thriving in temperate regions with well-drained soils, it occupies diverse habitats such as grasslands, hillsides, and riverbanks. For thousands of years, *A. argyi* has been extensively used in traditional Chinese medicine, both internally and externally, to treat conditions including dysmenorrhea, abdominal pain, and inflammation [36]. These therapeutic properties are largely attributed to rich bioactive compounds such as polysaccharides, flavonoids, alkaloids, and volatile oils in its leaves [37,38]. Glandular trichomes on *A. argyi* leaves serve as the primary sites for the synthesis and storage of volatile oils and are also key tissues involved in the production of flavonoids and terpenoids [35,39]. In recent years, however, the yield and quality of *A. argyi* have declined in cultivation areas due to environmental factors as well as long-term monocropping, genetic diversity loss, and improper management practices. Consequently, there is an urgent need to initiate genetic research aimed at identifying genes that could enhance the development of high-quality yields of *A. argyi* cultivars under stress conditions. Until now, *CAT* and *SOD* gene families have been reported in several plant species, but they have not been reported in *A. argyi*.

This study aimed to explore the *CAT* and *SOD* gene families in *A. argyi* using genome data, as well as by profiling the gene expression, cellular localization, and function through bioinformatics analysis [4,8,34,40]. Therefore, a genome-wide comprehensive investigation has been carried out to identify the 8 *CAT* and 14 *SOD* genes in *A. argyi* for the first time. Furthermore, their phylogenetic relationships, synteny analysis, gene structures, conserved motifs, cis-elements, gene ontology analysis, protein structure prediction, and functional annotation have been characterized. This provides a comprehensive basis for making the stress-resilient *A. argyi* transgenic through CRISPR/CAS9.

## 2. Results

### 2.1. Identification of CAT and SOD Gene Families in A. argyi

We performed a BlastP using query sequences of *AtCAT* and *AtSOD* genes against the *A. argyi* genome database. For result accuracy, we conducted the HMMER search and verified the *CAT/SOD* domains (PF00199, PF06628, PF00080, PF02777, and PF00081) using Pfam. A total of 22 *CAT/SOD* gene members were identified in the genome of *A. argyi* (Table 1). The identified genes members from the *CAT* and *SOD* gene families were lower in *B. napus*, *B. oleracea*, and *B. rapa* but higher than *A. thaliana*. Full-length protein sequences are also included in Table S1. There were 8 *CATs* gene members, identified on Chr09, Chr17, Chr02, Chr02, Chr09, Chr17, Chr02, and Chr10, and the protein length varied from the 466 (*AarCAT3*) to 543 (*AarCAT6*). The instability index ranged from 34.55 to 42.21, while the protein molecular weight also varied from 53.8 (*AarCAT3*) to 62.5 (*AarCAT6*) kDA, as per protein length. The aliphatic index of CAT proteins ranged from 70.12 to 74.68, showing stability over a wide temperature range and high aliphatic amino acid content. The isoelectric points (pI) ranged from 6.4 (*AarCAT4*) to 8.13 (*AarCTA6*), and only *AarCAT5* and *AarCAT6* were classified as acidic proteins (pI < 7). However, all that remained were basic proteins. The Grand Average of Hydropathicity (GRAVY) for all *CATs members* was negative, showing that *AarCATs* were hydrophilic proteins. Cellular localization of *AarCATs* was predicted to be peroxisomal (Table 1). The identified 14 *SOD* genes were categorized into 3 groups: *AarCSDs*, *AarFSDs*, and *AarMSDs*. These genes are present on Chr16, Chr0, Chr08, Chr09, Chr17, Chr10 and Chr01, while the protein length varied from 151 (*AarCSD5*) to 311 (*AarFSD2*, *AarFSD3*). The instability index ranged from 5 to 50.84, and the aliphatic index ranged from 66.96 (*AarFSD5*) to 93.81 (*AarFSD4*). Moreover, the molecular weights of the SOD protein ranged from the 15.5 (*AarCSD3*) to 35.6 (*AarFSD1*), while the isoelectric points (pI) ranged from 4.76 (*AarFSD2*) to 8.95 (*AarFSD1*). The Grand Average of Hydropathicity (GRAVY) for all *SODs members* was negative, except *AarCSD1*, *AarCSD2*, and *AarCSD4*. Cellular localization of *AarCSDs* was predicted in the chloroplast and cytoplasm, while the *AarFSDs* were predicted in the chloroplast, cytoplasm, and nucleus, and the *AarMSDs* were predicted in the mitochondria (Table 1).

### 2.2. Phylogenetic Relationships of CAT and SOD Genes and Chromosomal Distribution

**Figure 1:**
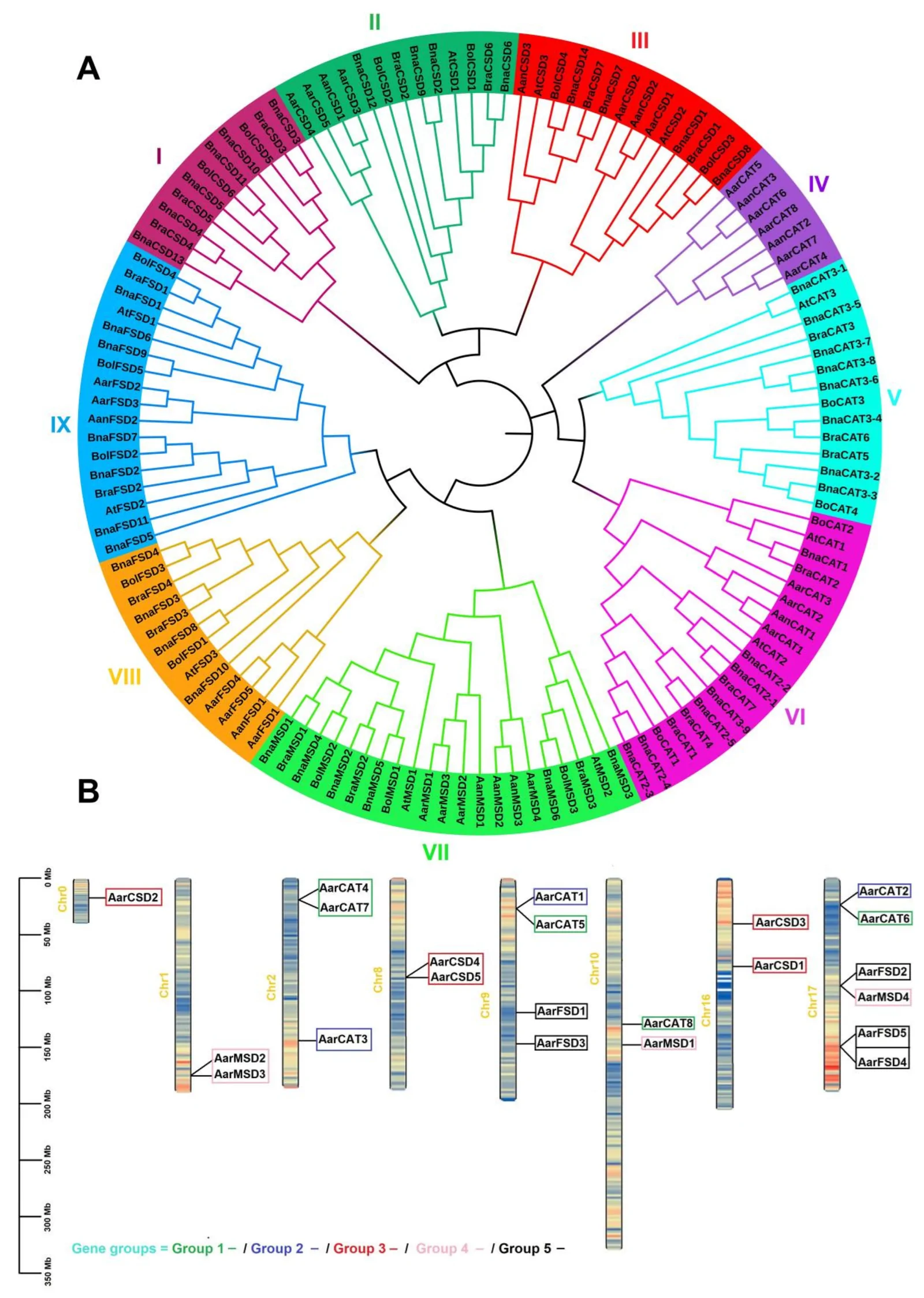
Phylogenetic relationships and chromosomal distribution. (**A**) Phylogenetic analysis of CAT and SOD proteins in *A. argyi*, *A. annua*, *B. napus*, *B. rapa*, *B. olerecea*, and *A. thaliana*. The phylogenetic tree was developed using the neighbor-joining method with 1000 bootstraps, using MEGA software. (**B**) The chromosome distribution mapping of *CAT* and *SOD* gene families in *A. argyi.*

Phylogenetic analysis was performed using the neighbor-joining method to examine the systematic evolutionary relationships of the *CAT* and *SOD* gene family in *A. argyi.* Protein sequences of CAT and SOD proteins were aligned using MEGA11 software between *A. thaliana*, *A. argyi*, *A. annua*, *B. napus*, *B. rapa*, and *B. olerecea.* In the phylogenetic tree, proteins were classified into nine groups (I–IX). *A. argyi* proteins were distributed across all the groups except groups I and groups V, as groups I and V were restricted with the *CAT/SOD* genes from *B. napus*, *B. rapa*, *B. olerecea*, and *A. thalina*, respectively. Group VII was the largest group, consisting of 21 gene members, and group IV only consisted of seven *A. argyi CAT* genes. Overall, groups I, II, III, V, VI, VIII, and IX consisted of 11, 13, 14, 14, 19, 13, and 17 gene members, respectively (Figure 1A).

Additionally, the pattern of *CAT/SOD* gene distribution reveals that the members of *A. argyi* and *A. annua* are closely related, which suggests a high homology rate between these species. Chromosomal localization analysis revealed the distribution of 22 *CAT/SOD* genes in *A. argyi*. The distribution of these genes across the chromosomes is not uniform. The *AarCSD2* gene was found on Chr0, *AarMSD2* and *AarMSD3* on Chr1, *AarCAT4*, *AarCAT7*, *AarCAT3* on Chr2, *AarCSD4* and *AarCSD5* on Chr8, *AarCAT1*, *AarCAT5*, *AarFSD1*, and *AarFSD3* on Chr9, *AarCAT8* and *AarMSD1* on Chr10, *AarCSD3* and *AarCSD1* on Chr16, and six gene members, including *AarCAT2*, *AarCAT6*, *AaFSD2*, *AarMSD4*, *AarFSD5*, and *AarFSD4*, were present on chromosome 17. Based on the gene linkages, they were divided into five groups (Figure 1B, Table S5).

### 2.3. Conserved Motifs, Conserved Structural Domains, and Gene Structure Analysis of the CAT and SOD Gene Families in A. argyi

**Figure 2:**
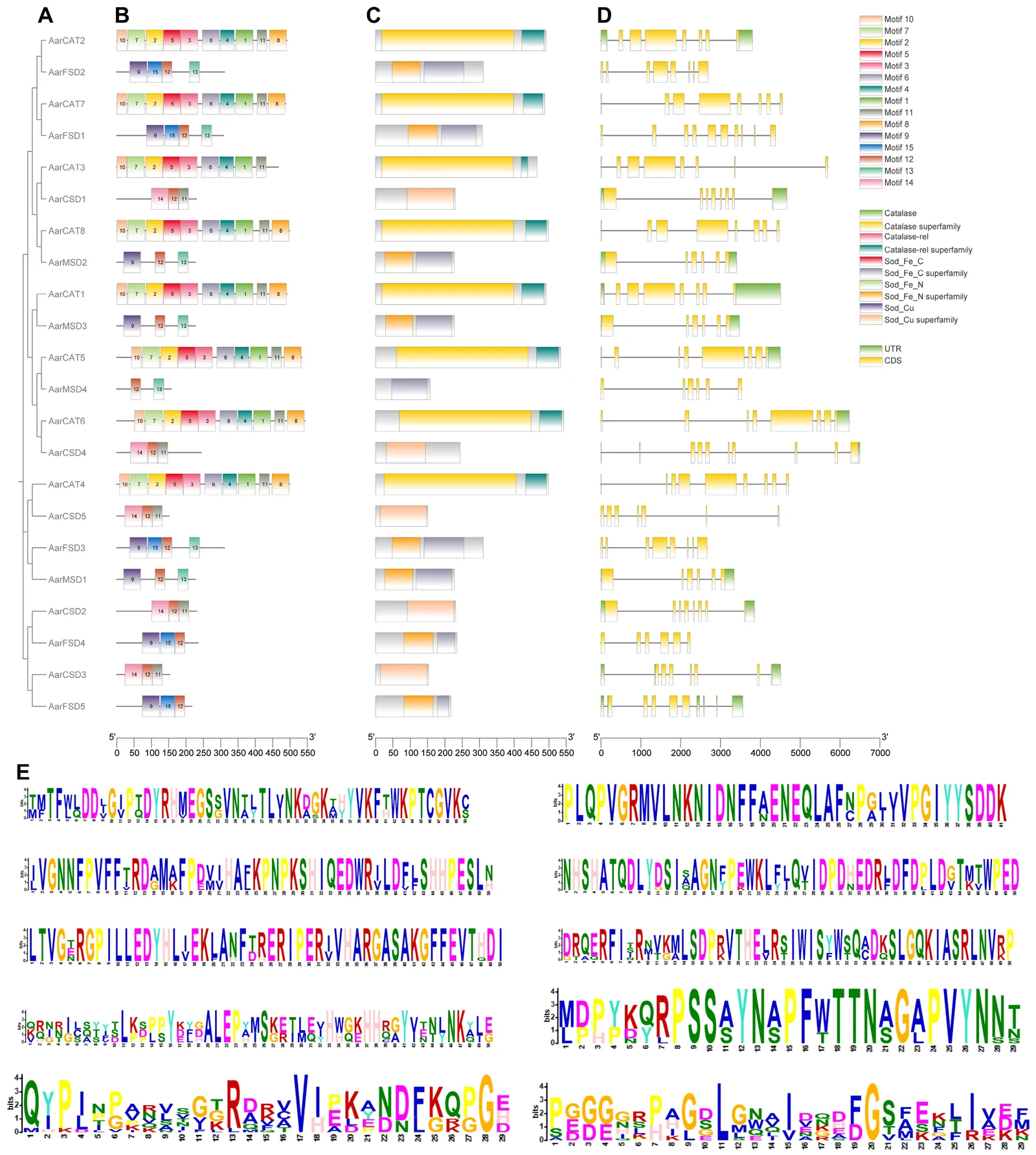
Phylogenetic relationship, conserved motif, and gene structure analyses. (**A**) Phylogenetic tree of the 22 analyzed proteins. (**B**) Distribution of conserved motifs. Boxes of 15 different colors represent different conserved motifs. (**C**) Prediction and analysis of conserved domains. (**D**) Exon/intron distribution, with yellow representing CDS and green representing UTRs. (**E**) Sequence logos displaying the highly conserved amino acid alignments for the identified typical motifs.

To further investigate the gene structure and conserved domains of *CAT/SOD* proteins, we analyzed 22 protein sequences through the MEME Suite and identified 15 conserved motifs across the *CAT* and *SOD* gene families, as shown in Figure 2. All AarCATs and AarSODs protein sequences from *A. argyi* contain the highly conserved CAT domains, whereas the AarFSDs, AarMSDs, and AarCSDs proteins also consist of Sod_Fe_C, Sod_Fe_N, and Sod_Cu domains (Figure 2B). Furthermore, the conservation patterns across the 22 proteins revealed specificity among their evolutionary clades and are strongly conserved within the groups.

The Gene structure of the *CAT* and *SOD* gene families was assessed by intron/exon structure analysis in *A. argyi*, which was performed to assess their diversity. The number of exons and overall gene length vary significantly between the different genes. The *AarCATs* genes generally exhibit longer and more complex genomic structures with multiple introns in some genes (*AarCAT6* and *AarCAT8*), while the *AarFSD*, *AarMSD*, and *AarCSD* gene members have shorter lengths and consist of few introns. Additionally, it was observed that specific genes across the families, including *AarCAT2*, *AarCAT7*, *AarCAT1*, *AarCSD1*, *AarCSD2*, *AarCSD3*, and *AarFSD5*, consist of untranslated regions (UTRs) at their extremities (Figure 2D).

Among the 22 proteins, 15 conserved motifs were identified. Most gene members of *CAT* and *SOD* consist of a similar number and type of motifs, with identical distribution patterns, suggesting that CAT and SOD proteins have strong functional similarities within their groups (Figure 2B). However, the *AarCAT* clade has a long-conserved chain of motifs (including motifs 13, 7, 2, 5, 3, 8, 4, 1, 11, and 6) that are totally different from the *AarSODs*, which possess their own specific motifs (motifs 9, 15, 12, and 10 for the *AarFSD/MSD* members). Furthermore, ten motif structures are similar between *AarCSD3* and *AarFSD5*, indicating that they have the same function. Moreover, the typical conserved domains and highly critical amino acid sequences related to the *CAT* and *SOD* gene family were mapped through motif sequence logos (Figure 2E), reinforcing the strict evolutionary conservation at the structural and amino acid levels. Motif sequence details are provided in the Supplementary Materials (Table S3).

### 2.4. Collinear Prediction and Sequence Similarity Analysis of AarCAT/SOD Genes

**Figure 3:**
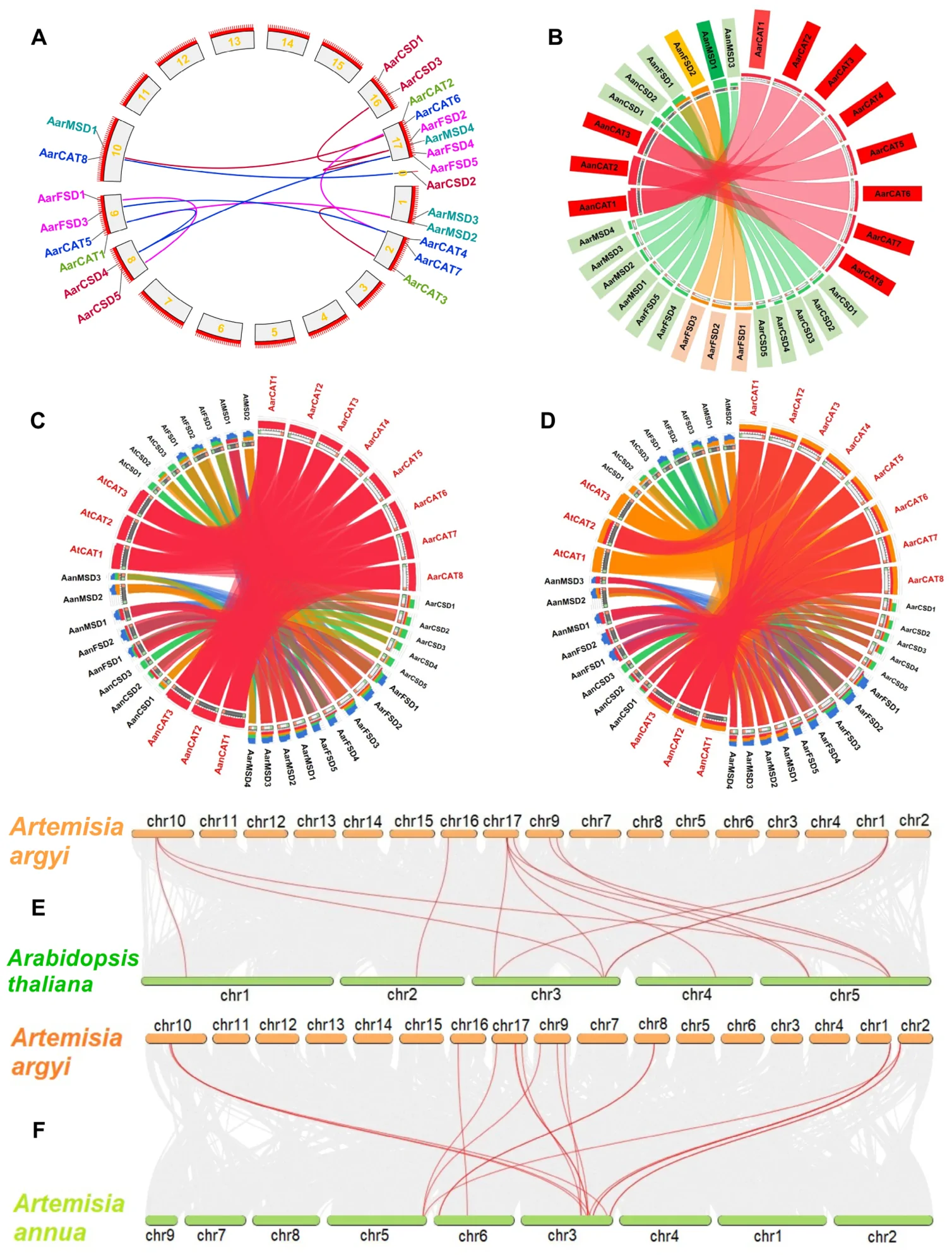
Collinear prediction and sequence similarity analysis of *CAT* and *SOD* gene families in *A. argyi*, *A. annua* and *A. thaliana.* (**A**): Mapping of collinear prediction of the *AarCAT*, *AarCSD*, *AarFSD*, and *AarMSD* family genes in *A. argyi*. Distinctly colored lines show intra-species paralogs, and the outer blocks indicate *A. argyi* chromosome numbers (1–17). (**B**,**D**): Visualizing sequence similarity and (**C**) e-value of the genes using the Circoletto online tool. Different colored ribbons represent varying levels of sequence similarity, with red denoting the highest level of sequence conservation. (**E**): Collinear prediction of the gene families between *A. thaliana* (chr 1–5) and *A. argyi* (chr 1–17). Red lines show the collinear events (orthologs) between the two species. (**F**): Collinear prediction of the gene families between *A. argyi* (chr 1–17) and *A. annua* (chr 1–9). Red lines show the high-density collinear events between the related *Artemisia* genomes, visualized by TBtools.

Evolution is critically caused by gene divergence and duplication events, which can also significantly promote the expansion of gene families and protein functions. In this study, intra-species collinear events were utilized to analyze the duplication of specific gene families (*AarCATs*, *AarCSDs*, *AarFSDs*, and *AarMSDs*) in the *A. argyi* genome (Figure 3A). Multiple collinear gene pairs were identified on 17 chromosomes, with duplication events visibly linking specific paralog genes such as *AarFSD1* with *AarFSD3* and *AarMSD1* with *AarMSD3*, at different chromosomal locations. These findings revealed that homologous genes may have similar functions, indicating functional diversity within these respective gene families in *A. argyi*.

Furthermore, sequence similarity of these *CAT* and *SOD* genes in *A. argyi*, *A. annua,* and *A. thaliana* was analyzed by Circoletto (Figure 3B). The dense network of red ribbons indicates genes’ highest sequence similarity (>99%), which is high among the *AarCAT* family members (*AarCAT1* through *AarCAT8*). Genes within the *AarCSD*, *AarFSD*, and *AarMSD* subfamilies are connected by green and orange ribbons, which indicate the intermediate-to-high homology (75% to 99% similarity) among themselves. Moreover, the multi-species sequence (Figure 3C,D) further illustrates the strong conservation of highly similar genetic profiles of *CAT* and *SOD* genes on the basis of e-value (Figure 3C) and sequence identity (Figure 3D) across different genomic datasets.

Additionally, we mapped the *A. argyi* genome against the *A. thaliana* genome and the closely related *A. annua*, respectively, and analyzed the collinear events of these genes to trace their evolutionary history (Figure 3E,F). *A. argyi* and *A. thaliana* genomes were compared, and a baseline number of orthologous pairs were identified, reflecting shared but distant ancestral lineages (Figure 3E). In contrast, synteny analysis between *A. argyi* and *A. annua* revealed a complex network of collinear events connecting the 17 chromosomes of *A. argyi* to the 3 chromosomes of *A. annua*. These findings implied a high degree of evolutionary conservation and genomic collinearity retention between the *Artemisia* species, suggesting that most of these orthologous genes resisted gene loss and were highly conserved following speciation and duplication processes.

### 2.5. Gene Ontology (GO) Analysis and Protein Interaction Prediction Analysis

**Figure 4:**
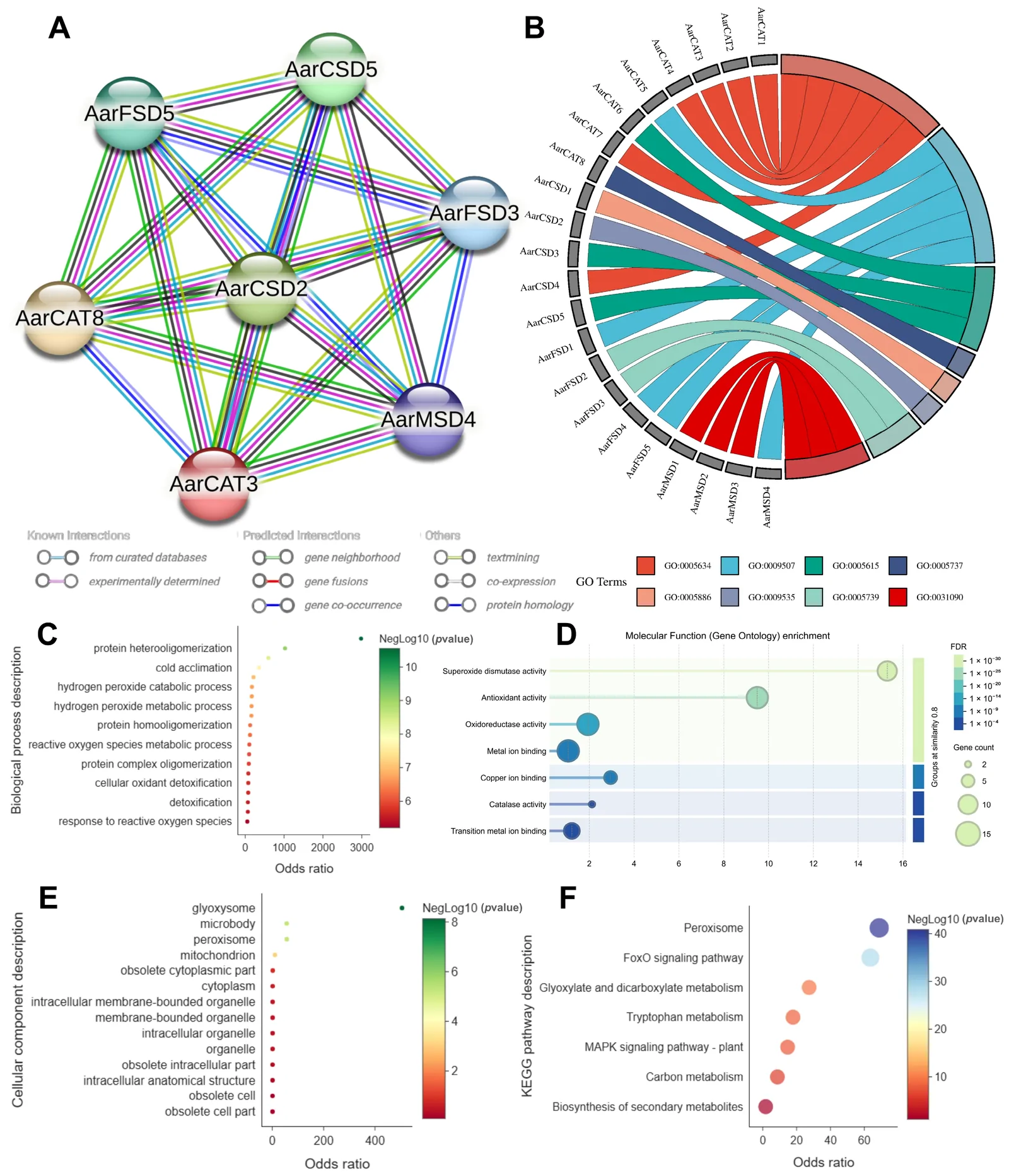
Protein interaction network, GO enrichment, and KEGG pathway analysis. (**A**): Protein–protein interaction (PPI) network predicted via STRING version 12.5 software, with colored lines representing different interaction evidence types (curated databases, experimentally determined). (**B**): Circos diagram illustrating the mapping between individual genes and specific GO functional categories. (**C**): Bubble plot of enriched Biological Process GO terms ordered by odds ratio. (**D**): Enrichment plot of Molecular Function GO terms showing gene counts and false discovery rate (FDR). (**E**): Bubble plot of enriched Cellular Component GO terms indicating subcellular localization. (**F**): Bubble plot of KEGG pathway enrichment analysis highlighting key metabolic and signaling pathways.

To deeply analyze the biological functions and protein–protein interaction networks of the *AarCATs* and *AarSODs* gene family members, protein–protein interaction (PPI) networks were predicted using STRING version 12.5. The interaction network revealed a highly interconnected core interactome consisting of key members, including *AarCSD2*, *AarCSD5*, *AarFSD3*, *AarFSD5*, *AarCAT3*, *AarCAT8*, and *AarMSD4*. These hub proteins are interconnected through multiple lines of evidence, including experimental determination, curated databases, gene co-occurrence, text mining, and co-expression patterns (Figure 4A). Gene GO mapping was represented by a circos plot, which mapped individual gene family members (*AarCAT*, *AarCSD*, *AarFSD*, and *AarMSD* genes) against key functional GO term categories, illustrating distinct, group-specific functional linkages (Figure 4B, Table S6).

To examine the functional attributes of the *AarCATs* and *AarSODs* genes, comprehensive GO annotation and KEGG pathway enrichment analyses were conducted. In the biological process enrichment, GO enrichment for biological processes revealed that the most significantly enriched processes, ordered by odds ratio, included protein heterooligomerization, cold acclimation, hydrogen peroxide catabolic process, and hydrogen peroxide metabolic process (Figure 4C). Molecular function enrichment analysis revealed that the functional activity and mapping confirmed that SOD activity and antioxidant activity were the most significantly enriched terms containing the largest gene counts (up to 15 genes). This was followed by oxidoreductase activity, metal ion binding, and copper ion binding (Figure 4D). Cellular component localization analysis showed that *AarCATs and AarSODs* members predominantly target the glyoxysome, microbody, and peroxisome, which exhibited the highest odds ratios (Figure 4E).

KEGG pathway enrichment analysis demonstrated that the candidate genes are involved in a critical metabolic process. The most prominent pathway was the peroxisome pathway, exhibiting both the highest odds ratio (approximately 60) and the most significant confidence score. Additional significant enrichment was identified in the FoxO signaling pathway, glyoxylate and dicarboxylate metabolism, and tryptophan metabolism, respectively (Figure 4F).

### 2.6. Analysis of the Structures, Phosphorylation Sites, and Signal Peptides of CAT/SOD Proteins in A. argyi

**Figure 5:**
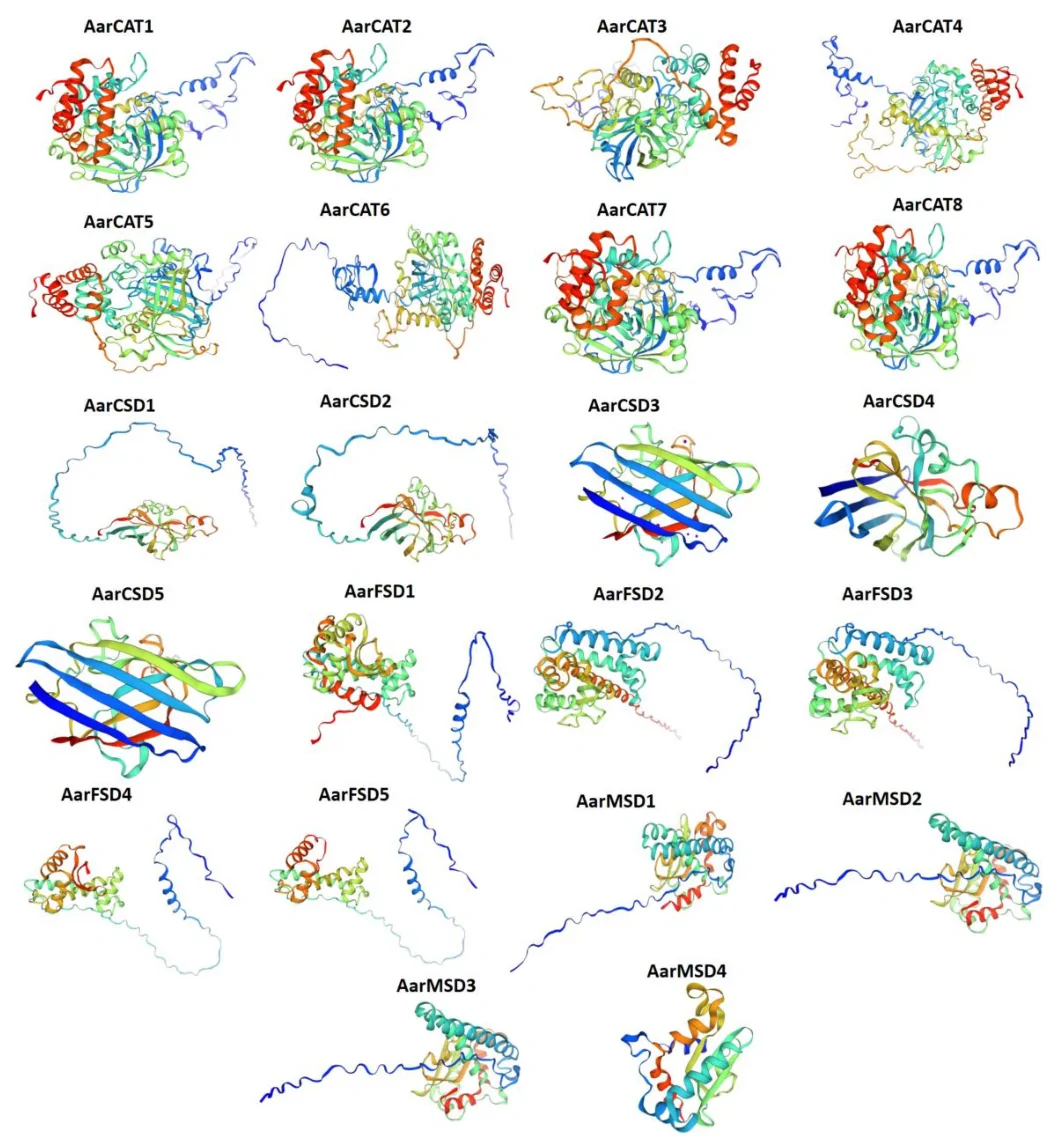
Homology modeling and 3D structures of the *CAT* and *SOD* families in *A. argyi*. The tertiary structures of 22 proteins across the *AarCAT*, *AarCSD*, *AarFSD*, and *AarMSD* gene subfamilies were generated. Specific structural domains are visible, including dense alpha-helical clusters characteristic of the *AarCAT*, *AarFSD*, and *AarMSD gene* families, prominent beta-barrel formations in *AarCSD3–5*, and extensive random coil regions present in distinct members such as *AarCSD1*, *AarCSD2*, and *AarFSD2*.

The structural characteristics of the *AarCAT* and *AarSOD* gene families were analyzed to establish a link between their structure and putative biological functions. The predictions of secondary structure revealed a composition of alpha-helices, extended strands, beta-turns, and random coils. To obtain deeper structural insights and validate these structural ratios, the 3D tertiary structures of all 22 candidate proteins were modeled (Figure 5). Secondary structure revealed a mix of α-helices, β-sheets, turns, and random coils. Within the CAT family, α-helix content ranged from 36.2% to 38.5%, β-sheets from 22.1% to 23.4%, turns from 7.9% to 8.5%, and coils from 31.1% to 32.5%. Among SOD subfamilies, the MSD proteins exhibited the highest α-helix content (~44%), whereas FSD proteins showed the lowest (~32%), while having the highest coil content (~36%). The complete information about protein composition is included in structural models that revealed highly conserved architectures within CAT and SOD subfamilies, but with divergence between them (Table S9). Post-translational modifications (PTMs) critically regulate protein activity and downstream functions. Phosphorylation mediates environmental stress signaling. NetPhos 3.1 predicted multiple phosphorylation sites in all members, indicating complex regulatory mechanisms.

The *AarCAT* members (*AarCAT1–8*) possess large, complex globular architectures characterized by dense clusters of alpha-helices and stable structural cores, which are essential for CAT enzymatic activity. Conversely, the *AarCSD* members have significant intra-family structural variation. *AarCSD3*, *AarCSD4*, and *AarCSD5* are prominently characterized by highly organized beta-barrel conformations formed by anti-parallel extended beta-strands, a hallmark structural motif of copper/zinc superoxide dismutases. In contrast, *AarCSD1* and *AarCSD2* present much smaller folded cores heavily dominated by extended, unstructured random coils. The *AarFSD* (*AarFSD1–5*) and *AarMSD* (*AarMSD1–4*) members display remarkably similar genetic organization in their 3D folding, predominantly composed of alpha-helical bundles. Notably, several members within these groups, particularly *AarFSD1*, *AarFSD2*, *AarFSD3*, and *AarMSD2*, possess highly elongated, unstructured random coil tails extending from their structured catalytic domains, which may facilitate flexible protein–protein interactions.

In addition, this structural evidence strongly suggests that these specific CAT/SOD proteins in *A. argyi* are not secreted proteins but are instead synthesized and retained intracellularly to function within specific subcellular localization.

### 2.7. Promoter cis-Acting Element Analysis

**Figure 6:**
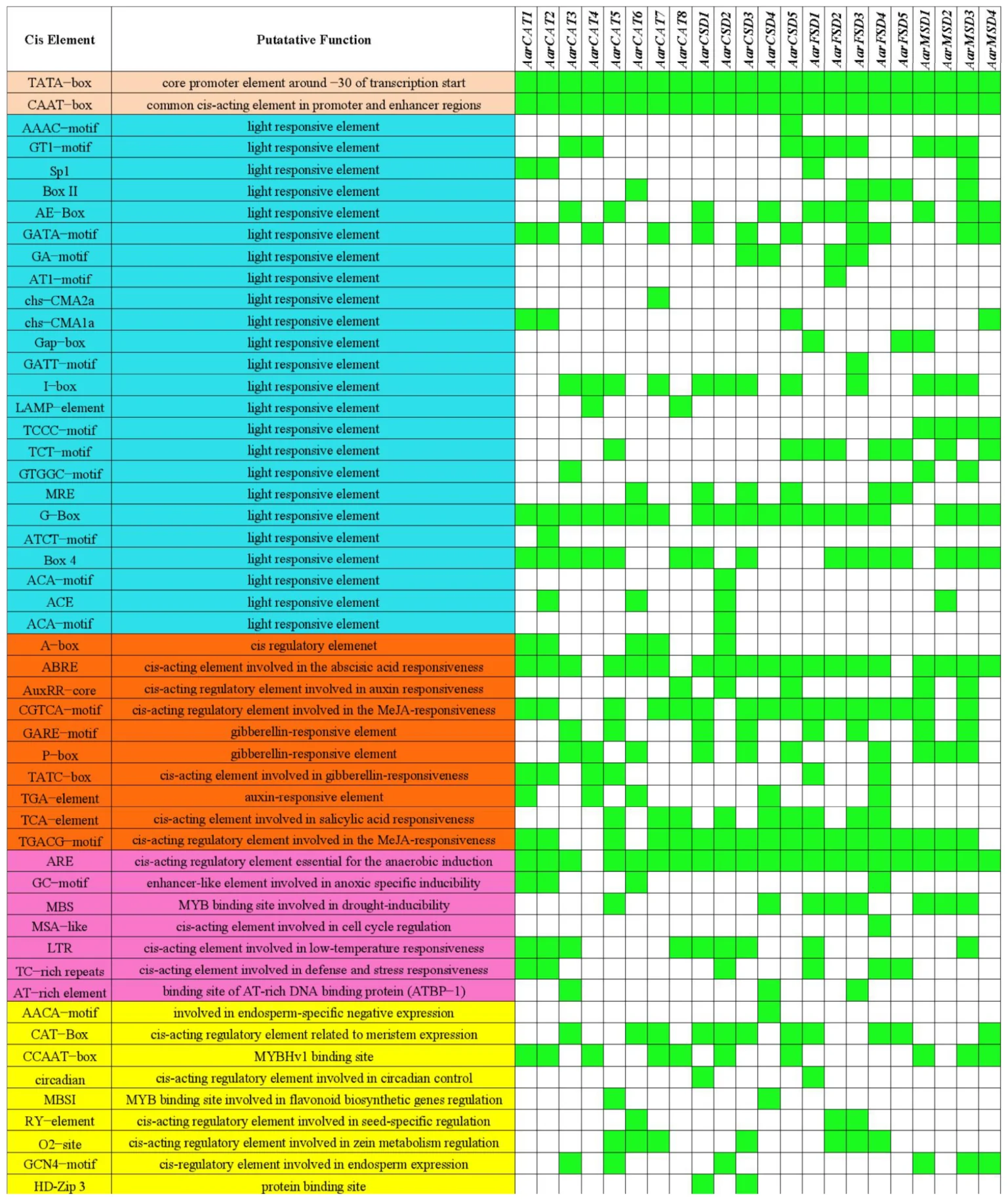
Promoter *cis*-acting element analysis of the *CAT* and *SOD* gene families in *A. argyi*. On the left, *cis*-elements including core promoter, light, phytohormone, abiotic stress, and growth/development regulation responsive elements are represented by five different categorical colors (tan, blue, orange, pink, and yellow, respectively). On the right, the green squares indicate the presence of a specific *cis*-element in the promoter region, while the blank (white) squares demonstrate the absence of the *cis*-element.

To understand transcriptional regulation and potential environmental responsiveness, *cis*-regulatory elements were analyzed in the promoter regions of *A. argyi* using the PlantCARE database. A total of 52 *cis*-elements were identified and categorized into five distinct functional groups, including core promoter elements (2), light-responsive elements (24), phytohormone-responsive elements (10), abiotic stress-responsive elements (7), and plant growth and development regulation elements (9) (Figure 6).

Core promoter transcriptional elements, specifically the TATA-box and CAAT-box, were universally found across all 22 analyzed genes, confirming standard fundamental promoter elements. Light-responsive elements contain the largest regulatory group; notably, the G-box motif was highly common, being present in over 90% of the genes and absent only in *AarCSD4* and *AarFSD4*. Other critical light-responsive elements, such as Box 4 and the AE-box, were also widely distributed across the *CAT* and *SOD* genes.

In hormone signaling, abscisic acid (ABA)-responsive elements (ABRE) were detected throughout the gene families, with specific exceptions only in *AarCAT3*, *AarCAT7*, *AarCAT8*, *AarFSD1*, *AarFSD3*, *AarFSD4*, *AarMSD1*, and *AarMSD2*. Additionally, MeJA-responsive elements (CGTCA-motif and TGACG-motif) were widely mapped across numerous genes. Other hormone-directed motifs, including salicylic acid-responsive (TCA-element), gibberellin-responsive (GARE-motif, P-box, and TATC-box), and auxin-responsive elements (TGA-element, AuxRR-core), were also well-represented. Furthermore, environmental stress adaptation motifs were prominently identified; elements essential for anaerobic induction (ARE) were highly prevalent, alongside critical motifs involved in low-temperature responsiveness (LTR), drought inducibility (MBS), and general defense and stress responsiveness (TC-rich repeats) (Table S7).

### 2.8. Comprehensive Expression Profiling, Subcellular Localization, and Stress Responsiveness of CAT and SOD Genes in A. argyi

**Figure 7:**
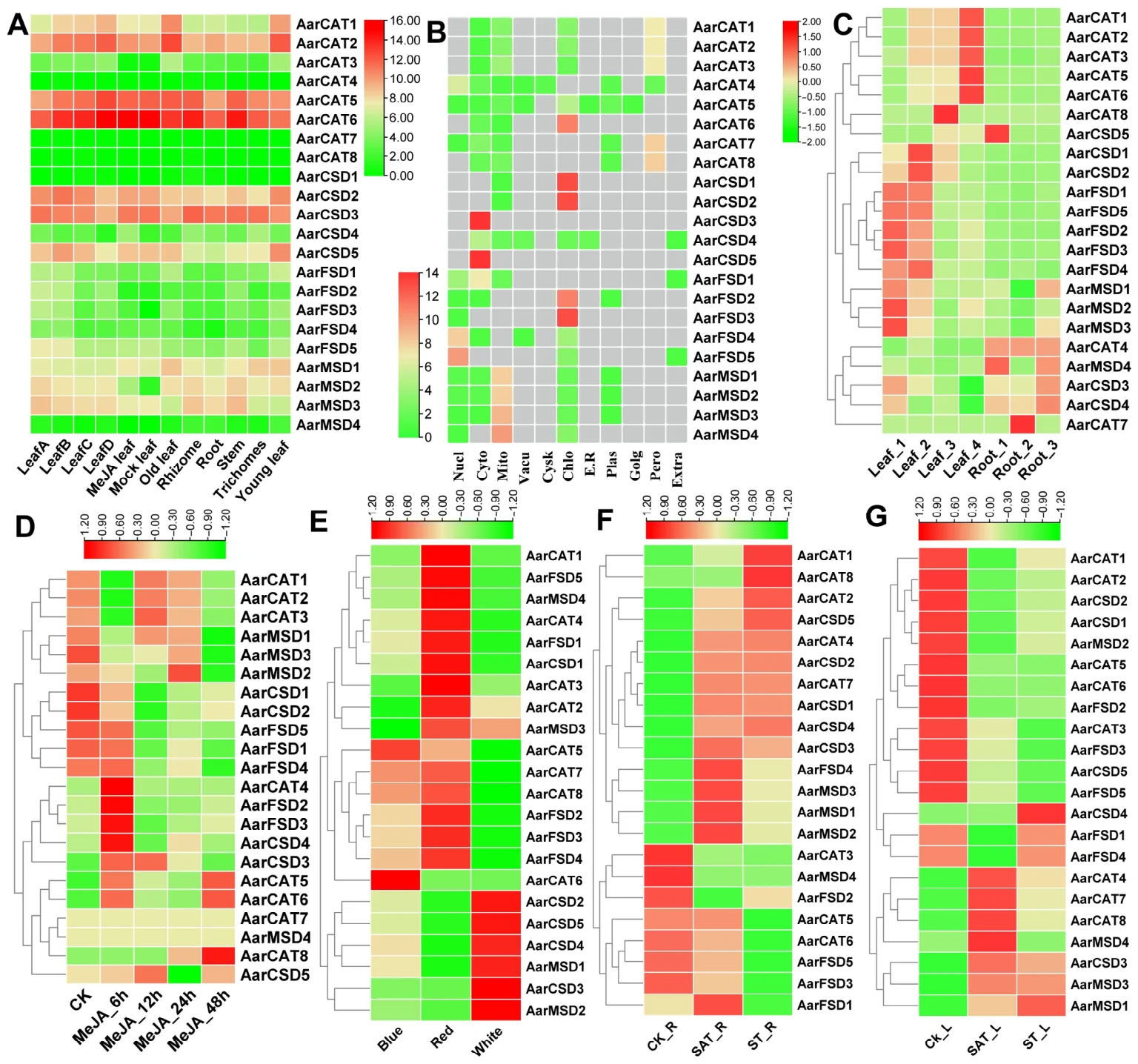
Comprehensive expression profiling, subcellular localization, and environmental responses of *CAT* and *SOD* gene families in *A. argyi*. (**A**) Heatmap across diverse tissues (leaves, stems, roots, rhizomes, and trichomes) and MeJA/mock treatments. (**B**) Subcellular localization distribution mapping across major cellular organelles and compartments (Nucl, Cyto, Mito, Vacu, Cysk, Chlo, E.R, Plas, Golg, Pero, and Extra). (**C**) Comparative expression analysis between leaf and root tissues. (**D**) Expression profiles under MeJA treatment (CK, 6 h, 12 h, 24 h, and 48 h). (**E**) Transcriptional modulation under different light qualities (blue, red, and white). (**F**) Expression profiles in roots under control (CK_R), saline–alkali treatment (SAT_R), and salt treatment (ST_R). (**G**) Expression profiles in leaves under control (CK_L), saline–alkali treatment (SAT_L), and salt treatment (ST_L). Legend value showing higher scores indicates greater prediction confidence.

To determine the physiological roles, regulatory expressions, and cellular localization of the *CAT* and *SOD* gene families in *A. argyi*, comprehensive expression profiling was performed across diverse tissues, developmental stages, light qualities, hormonal treatments, and abiotic stress conditions (Figure 7).

Expression profiling across various organs and developmental stages, including young leaves, old leaves, stems, roots, rhizomes, and trichomes, was performed for the *CAT* and *SOD* gene families (Figure 7A). The *AarCAT* and *AarSOD* gene families have a broad range of expression across vegetative tissues, whereas *AarCAT1*, *AarCAT2*, *AarCAT5*, *AarCAT6*, *AarCSD2*, *AarCSD3*, *AarCSD5*, *AarMSD1*, *AarMSD2* and *AarMSD3* showed high expression in 12 organs, including leaf A, leaf B, leaf C, leaf D, MeJA leaf, Mock leaf, Old leaf, rhizome, root, stem, trichomes, and young leaf (Figure 7A, Table S8).

The subcellular localization of the *AarCATs* and *AarSODs* genes was checked across major cellular compartments, including the nucleus, cytoplasm, mitochondria, vacuole, cytoskeleton, chloroplast, endoplasmic reticulum, plasma membrane, Golgi apparatus, peroxisome, and extracellular spaces, to correlate gene function with cellular components (Figure 7B, Table S4). The distribution pattern illustrates that members are strategically localized to manage ROS produced by metabolic pathways. Notably, specific *AarCATs* and *AarSODs* showed prominent localization targeting peroxisomes, chloroplasts, mitochondria, cytoplasm, and nucleus, aligning with their significant roles in mitigating oxidative stress. *AarCAT* and *AarSOD* gene regulation was compared between leaf and root, and results revealed that most genes were upregulated in Leaf_2 compared with Root_2 transcriptomic data, while *AarCAT1* and *AarCAT6* showed upregulation (Leaf_2,3,4) and downregulation in root (Root_1,2,3) transcriptome (Figure 7C).

We measured the *AarCAT*, *AarCSD*, *AarFSD*, and *AarMSD* genes that responded to methyl jasmonate (MeJA) at five time points: 0 h (control), 6 h, 12 h, 24 h, and 48 h. (Figure 7D). The heatmap illustrates that MeJA triggers extensive transcriptional regulation. Several *AarCAT/SOD* genes, particularly within the *AarCAT* and *AarSOD* gene families, exhibited time-dependent induction, showing higher expression in MeJA_6 h, MeJA_24 h, and MeJA_12 h, respectively (Figure 7D). The *AarCAT1*, *AarCAT2*, and *AarCAT3* bar graphs showed significant expression patterns at 6 h and 12 h post-treatment. After 24 h, the genes showed recovery and were sustained through 48 h. Moreover, *AarCAT5* and *AarCAT6* also showed an early stress response at 6 h and 12 h. The *AarSOD* gene family displayed moderate expression fluctuations overall in different time intervals. While *AarCSD1* and *AarFSD5* showed downregulation at specific time intervals, their expression levels showed recovery at 24 h and were sustained for 48 h. In contrast, the *AarMSD2* and *AarCSD3* genes particularly maintained high and consistent expression without significant deviation across different time intervals (Figure S1).

Under different light qualities, the expression profiles of *AarCAT* and *AarSOD* gene families significantly changed, as light highly influenced the transcriptional activity of these genes (Figure 7E). Under different light qualities such as blue, red, and white light, most genes from *AarCAT/SOD* were significantly upregulated under red light and downregulated under white light, except *AarCSD2-AarMSD2.* There is a strong connection between harvest light, transport of photosynthetic energy, and enzyme use to detoxify reactive oxygen in *A. argyi.* Under red light, different genes from the *CAT* gene family (*AarCAT1*, *AarCAT2*, and *AarCAT3*) and *SOD* gene family (*AarFSD1*, *AarFSD2*, *AarFSD3*, and *AarFSD5*) showed significant upregulation as compared with both blue and white lights. However, under blue light, the expression levels of the *AarCAT5* and *AarCAT6* genes were significantly higher when compared with red and white light. Under white light, the expression levels of the *AarFSD* and *AarCAT* genes were relatively low, except for *AarCSD4*, which showed a higher expression level under white light.

However, the *AarMSD* family (*AarMSD1*, *AarMSD2*, and *AarMSD3*) showed regulatory stability across all the light qualities, suggesting that photoreceptor-mediated signaling does not regulate the mitochondrial ROS-scavenging functions of these genes (Figure S2).

### 2.9. Transcriptional Regulation Under Salt and Saline–Alkali Stresses

To investigate the role of *AarCAT* and *AarSOD* genes in osmotic and ionic resilience, expression profiles were analyzed under salt (ST) and saline–alkali (SAT) treatments in both roots (Figure 7F) and leaves (Figure 7G). Both stress conditions induced significant differential expression across the gene families. In roots (Figure 7F), the *AarCAT/SOD* genes showed upregulation in response to SAT and ST, reflecting an adaptive defense response to neutralize stress-induced oxidative damage in root tissues. However, in leaf tissues, *CAT/SOD* genes were mostly downregulated to sustain cellular redox homeostasis under high salinity and alkalinity (Figure 7G). In leaf tissues, the transcriptional regulation of *AarCAT5 (~1.7-fold)*, *AarCAT6 (~2-fold)*, *AarCSD1 (~3.2-fold (SAT)* and *~1.8-fold (ST))*, and *AarCSD2 (~2.3-fold* in SAT) genes were significantly downregulated under both stresses as compared to the control (CK). Conversely, the *AarCAT4 ~112-fold (SAT)*, *~62-folod (ST)*, *AarCAT7*, *AarCAT8 ~509-fold (SAT)*, *~225-fold (ST)*, *AarCSD3 ~1.3-fold* genes were significantly upregulated and induced by both ST and SAT stresses, while the *AarCAT3 (~0.97-fold)* and *AarCSD4 (~1.46-fold)* genes showed significantly high expression under salt stress to counter the stress-induced ROS in leaves (Figure S3).

In the *A. argyi* root, *CAT* gene family members, including *AarCAT1 (~1.1, ~1.4)* and *AarCAT2 (~1.4, ~1.6*), showed high expression levels under SAT and ST as compared with the control (T1), respectively. The expression level of *SOD* gene family members, including *AarCSD1 (~1.35, ~1.34)*, *AarCSD2 (~1.51, ~1.53)*, *AarCSD3 (~1.33, ~1.26)*, *AarCSD4 (~1.74, ~1.87)*, *AarMSD1 (~2.62, ~1.73)*, *AarMSD2 (~2.24, ~1.55)*, and *AarMSD3* (~1.81, ~1.38), showed significantly high expression levels under both SAT and ST stresses, respectively, as compared with the control condition (T1).

In contrast, roots and leaves showed distinct *SOD* gene member expression patterns. The *AarCAT* genes, including *AarCSD2*, *AarCSD3*, and *AarCSD4*, were mostly activated in leaves to mitigate the stress response. However, *AarCAT* and *AarSOD* gene family members were equally activated in roots to regulate stress. Members including *AarCSD1*, *AarCSD2*, *AarCSD3*, *AarCSD4*, *AarMSD1*, *AarMSD2*, and *AarMSD3* showed significantly higher transcript levels under SAT and ST stresses, respectively. Unlike the *AarSOD* genes, the root *CAT* genes (*AarCAT1*, *AarCAT3*, *AarCAT5*, and *AarCAT6*) maintained expression in roots under both stresses, contrasting with the responsive *SOD* genes. This indicates independent regulatory mechanisms for the two antioxidant families in this tissue (Table S10).

### 2.10. Growth Attributes and ROS Accumulation, Photosynthetic and Gaseous Exchange Rate

**Figure 8:**
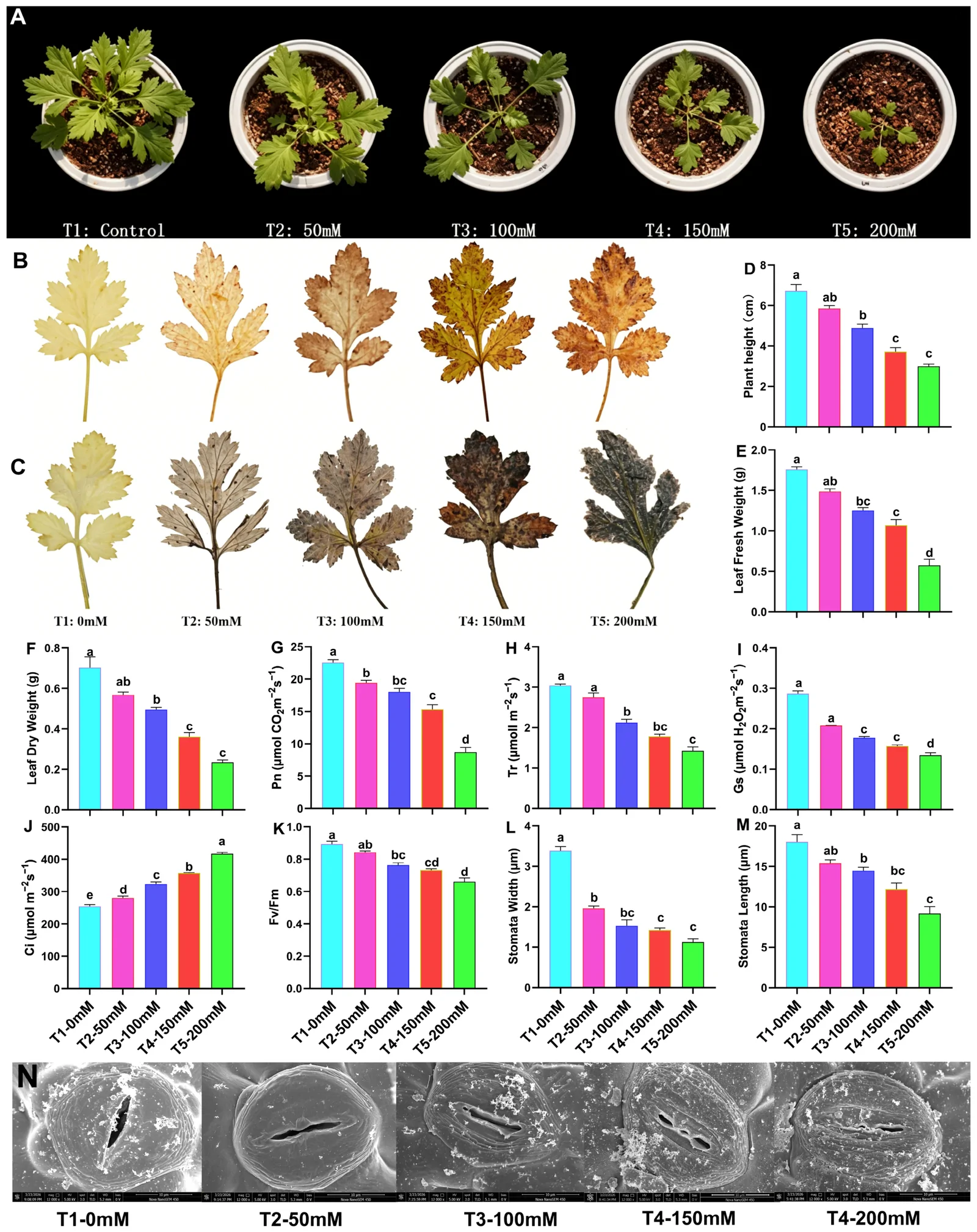
Morphological, physiological, and stomatal responses to stress treatments. (**A**) Phenotypic observations of *A. argyi* under varying salt stress treatments (T1: 0 mM/Control, T2: 50 mM, T3: 100 mM, T4: 150 mM, and T5: 200 mM). (**B**,**C**) Histochemical (DAB/NBT) staining of stress-induced ROS accumulation in leaves across the stress treatments. (**D**–**F**) Key morpho-physiological growth parameters, including plant height (**D**), leaf fresh weight (**E**), and leaf dry weight (**F**). (**G**–**J**) Variations in leaf gas exchange attributes under salt stress, presenting net photosynthetic rate (Pn) (**G**), transpiration rate (Tr) (**H**), stomatal conductance (gs) (**I**), and intercellular CO_2_ concentration (Ci) (**J**). (**K**) Impact of stress treatments on the maximal quantum yield of photosystem II (Fv/Fm). (**L**,**M**) Stomatal dimensions, detailing stomatal width (**L**) and stomatal length (**M**). (**N**) High-resolution scanning electron microscopy (SEM) illustrating the stomatal structure, pore aperture, and guard cell architecture of stomata under stress conditions. Distinct lowercase letters above the error bars denote statistically significant differences between treatment groups at *p* < 0.05.

The morpho-physiological traits revealed a significant decrease in plant growth and an increase in ROS in leaves. Under normal conditions, plants showed significant growth, which decreased across the stress treatments (Figure 8A). Plant height was significantly reduced across treatments, with values of 13.0% (T2), 27.2% (T3), 44.8% (T4), and 55.4% (T5), as compared with the control (T1) (Figure 8A,D). Moreover, leaf fresh weight decreased by 15.4%, 28.7%, 39.1%, and 67.2%, and dry weight reduced by 19.2%, 29.5%, 48.7%, and 66.5% for T2–T5 treatments, respectively (Figure 8E,F). Salt stress significantly reduced the photosynthetic efficiency. Net photosynthesis (Pn) (13.9%, 20.1%, 32%, and 61.3%), transpiration (Tr) (9.6%, 30.1%, 41.6%, and 53.1%), stomatal conductance (Gs) (27.5%, 38.1%, 45.3%, and 53.2%), and Fv/Fm (5.6%, 14.4%, 18%, and 25.8%) were decreased, and intercellular CO_2_ concentration (Ci) (10.3%, 27.1%, 40.1%, and 63.7%) was increased in T2-T5 treatments, as compared with the control (T1) (Figure 8G–K). Salt stress ruptured the stomatal structure; stomatal length (14.7%, 19.8%, 32.5%, and 49.1%) and stomatal width (42%, 54.7%, 57.8%, and 66.%) were reduced in T2–T5 treatments as compared with the control treatment (T1) (Figure 8L,M). These effects caused changes in the turgidity and conductance of stomata for gaseous exchange and photosynthetic efficiency (Figure 8N). Under microscopy, stomata showed progressive reductions in both length and width as stress levels increased, consistent with a response that restricts water loss. NBT and DAB staining revealed ROS activity in leaves under stress conditions. ROS increased with increasing salt stress levels, and accumulation of ROS was observed in leaves through DBA and NBT staining (Figure 8B,C). This staining showed the critical breakdown in cellular redox homeostasis and massive oxidative damage in plants under salt stress. Consistent with the growth reduction, our results indicate the functional impairment of the photosynthetic electron transport chain and sustained photoinhibition.

### 2.11. Salt Stress Impact on Photosynthetic Pigments, Osmotic Adjustment, and Activation of Antioxidant Defense Systems

**Figure 9:**
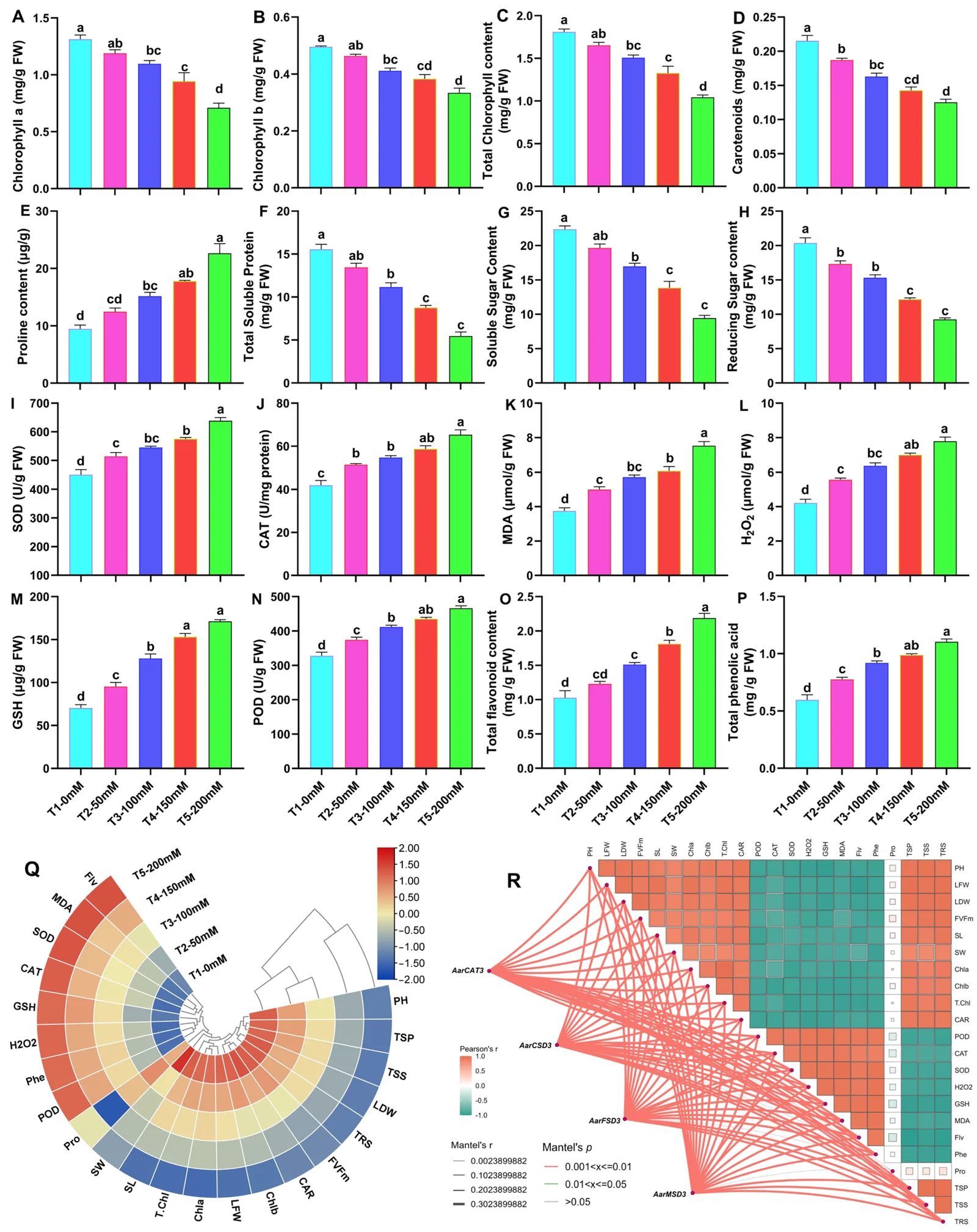
Physiological, biochemical, and antioxidant responses of *A. argyi* under different stress treatments and their associations with selected *AarCAT* and *AarSOD* genes. (**A**) Chlorophyll a content; (**B**) chlorophyll b content; (**C**) total chlorophyll content; (**D**) carotenoid content; (**E**) proline content; (**F**) total soluble protein content; (**G**) soluble sugar content; (**H**) reducing sugar content; (**I**) superoxide dismutase (SOD) activity; (**J**) catalase (CAT) activity; (**K**) malondialdehyde (MDA) content; (**L**) hydrogen peroxide (H_2_O_2_) content; (**M**) reduced glutathione (GSH) content; (**N**) peroxidase (POD) activity; (**O**) total flavonoid content; (**P**) total phenolic acid content; (**Q**) circular heatmap and hierarchical clustering showing variation in morphological, photosynthetic, osmotic-adjustment, antioxidant, ROS, and secondary-metabolite traits among the different stress treatments; (**R**) Mantel correlation network showing associations between the expression of selected *AarCAT* and *AarSOD* genes (*AarCAT3, AarCSD3, AarFSD3,* and *AarMSD3*) and physiological–biochemical traits. In the network, line thickness represents the strength of Mantel’s *r*, while line color indicates statistical significance; the Pearson correlation matrix among physiological traits is shown in the background. Different lowercase letters above bars indicate significant differences among treatments (*p* < 0.05). Abbreviations: PH, plant height; LFW, leaf fresh weight; LDW, leaf dry weight; F_v_/F_m_, maximum quantum yield of photosystem II; SL, stomatal length; SW, stomatal width; Chla, chlorophyll a; Chlb, chlorophyll b; T.Chl, total chlorophyll; CAR, carotenoids; POD, peroxidase; CAT, catalase; SOD, superoxide dismutase; H_2_O_2_, hydrogen peroxide; GSH, reduced glutathione; MDA, malondialdehyde; Flv, flavonoids; Phe, total phenolic acids; Pro, proline; TSP, total soluble protein; TSS, total soluble sugar; TRS, total reducing sugar. Different lowercase letters above the bars indicate significant differences among treatment means according to the least significant difference (LSD) test at *p* < 0.05. Bars sharing at least one letter are not significantly different, whereas bars with no letter in common differ significantly.

Salt stress causes a significant decline in the photosynthetic pigments and their performance. Photosynthetic pigments, including chlorophyll a (9.3%, 16.4%, 28.1%, and 45.9%), chlorophyll b (6.5%, 17%, 22.7%, and 32.6%), total chlorophyll (8.6%, 16.6%, 26.6%, and 42.3%), and carotenoid (13.1%, 24.3%, 33.7%, and 41.7%), decreased along with increased stress conditions in T2-T5 as compared with the control (T1). However, to evaluate the osmotic adjustment capacity of *A. argyi* under salt stress, osmolyte accumulation and primary metabolites were measured. Proline content is a critical stress-responsive osmoprotectant and was increased by 31.7%, 60.5%, 88%, and 139.6% in T2–T5 during salt treatments, respectively. This metabolic regulation indicates a massive reallocation of energetic resources away from basic growth and primary metabolism toward the synthesis of specific stress-defense compounds like proline. Furthermore, total soluble protein content (13.4%, 28.2%, 43.6%, and 64.8%), soluble sugar content (12.1%, 24.1%, 38.1%, and 57.8%), and reducing sugar content (15%, 24.8%, 40.2%, and 54.6%) decreased gradually with the salt intensity in T2–T5 treatments compared with the control (T1). In contrast, primary carbohydrates and proteins were severely depleted by the salt stress (Figure 9A–H).

Salt treatments triggered a substantial oxidative stress response, measured by ROS (H_2_O_2_ and MDA) and lipid peroxidation assays. MDA content and H_2_O_2_ levels increased progressively with increasing stress levels, as compared to the control treatment (T1) (Figure 9P,Q). This indicates oxidative damage associated with lipid peroxidation. Moreover, MDA content (33.2%, 52.5%, 62.1%, and 100.8%), and H_2_O_2_ (31.9%, 50.7%, 65.9%, and 84.8%) increased along with salt stress in T2–T5 as compared to the control (T1). To mitigate the oxidative stress, the plants produced a comprehensive antioxidant defense response. Therefore, SOD (14%, 20.8%, 27.4%, and 41.6%), CAT (22.4%, 30.3%, 39.7%, and 55.6%), and POD (14.2%, 25.6%, 32.8%, and 42.3%) enzymatic activities increased with salt stress intensity from 50 mM to 200 mM, respectively, when compared with the control (T1-0 mM). However, non-enzymatic antioxidants such as GSH (35.4%, 81.9%, 117.6%, and 143.4%), total flavonoids (19.8%, 47%, 76.6%, and 112.9%), and total phenolic acid content (29.9%, 54%, 65.4%, and 84.7%) also increased progressively with increased salt stress from 50 mM to 200 mM, as compared with the control (0 mM-salt) (Figure 9).

Hierarchical clustering heatmap and Mantel plot network analyses were used to illustrate the results of physiological traits and their correlation with the *CAT* and *SOD* gene families. The heatmap is divided into two clusters. One cluster, comprising ROS (MDA, H_2_O_2_) alongside both enzymatic (SOD, POD, and CAT) and non-enzymatic (GSH, flavonoids, phenolics, and Proline) antioxidants, was highly associated with the T4-150 mM salt treatment and T5-200 mM salt treatment. In contrast, growth parameters and photosynthetic pigments formed a separate cluster and were reduced under the same conditions.

## 3. Discussion

A genome-wide analysis identified 22 antioxidant genes in *A. argyi*, including 8 *AarCAT* and 14 *AarSOD* genes. The *SOD* genes comprised *CSD*, *FSD*, and *MSD* subfamilies [2,14]. The number of *CAT/SOD* genes in *A. argyi* was intermediate between *Brassica* species and *Arabidopsis thaliana*, suggesting specific evolutionary duplication and retention events linked to the *Artemisia* lineage [4,12,13,14]. Synteny and collinearity analyses revealed orthologous relationships between *A. argyi* and *A. annua* across the 17 chromosomes, indicating a high degree of evolutionary conservation and resistance to gene loss. The biological functions of these antioxidant enzymes are strictly regulated by their structural architectures. Protein structure predictions for *AarCAT* genes presented dense alpha-helical features forming complex globular cores essential for catalytic stability, while *AarCSD* genes displayed highly organized beta-barrel conformations typical of Cu/Zn SODs. The aliphatic index (AI) of CAT/SOD protein showed the thermostability of proteins, ranging from 66.96 to 93.81 across all family members. Notably, SOD proteins exhibited significantly higher AI values (average ~84.0) compared to CAT proteins (average ~72.6), with the *AarCSD4* protein showing the highest AI (93.81). This suggests that SOD proteins are relatively more thermostable than CATs, which is advantageous for maintaining ROS-scavenging activity under adverse environmental conditions. Furthermore, negative GRAVY scores confirm that these proteins are highly hydrophilic. Predicted subcellular localizations included the peroxisome, chloroplast, mitochondria, and cytoplasm sites in the cell, which are directly associated with metabolic ROS production [6,20,22]. These proteins were predicted to localize to organelles where ROS are produced. The capacity of *A. argyi* to adapt to environmental fluctuations is heavily dependent on rapid transcriptional reprogramming. Promoter analysis revealed ABRE, MeJA, ARE, and MBS elements in *AarCAT* and *AarSOD* genes, highlighting complex signaling pathways [4,9,12]. Consistent with these findings, MeJA treatment induced their expression within an early time period of 6–12 h. Additionally, red light upregulated several of these genes, suggesting possible coordination between light signals, photosynthetic energy transport, and antioxidant gene expression. Notably, *AarMSD* members maintained stable expression across different light qualities, implying that mitochondrial ROS scavenging operates independently of this photoreceptor regulation. Osmotic and ionic stress reduced plant growth and impaired physiological function. Salt stress severely reduced plant height, leaf fresh weight, and leaf dry weight (Figure 8D–F) [3,7]. At the structural level, stomatal length and width decreased with increasing salt concentration, reflecting a gradual degradation of the stomatal architecture. Consequently, stomatal conductance, transpiration rate, and photosynthetic efficiency (Pn and Fv/Fm) also declined under T2-T5 stress treatments, respectively [7]. The reduction in photosynthetic pigments is consistent with impaired thylakoid membrane function; chlorophyll a and total chlorophyll decreased by up to 45.9% and 42.3%, respectively, under 200 mM NaCl stress conditions, as compared with the control [41]. To survive this stress, *A. argyi* executes a drastic metabolic shift. Soluble proteins, soluble sugars, and reducing sugars decreased with increasing salinity, reflecting a severe depletion of primary metabolites [42,43]. In contrast, proline content increased by 139.6% at 200 mM NaCl compared to the control treatment. This indicates a strategic reallocation of energetic resources away from basic vegetative growth and into the biosynthesis of the specific osmoprotectants required to maintain cellular turgidity. Despite this osmotic adjustment, high salinity triggered a severe oxidative burst, visually confirmed in situ by NBT and DAB histochemical staining [7,44]. H_2_O_2_ and MDA levels progressively increased with salinity, indicating critical breakdowns in redox homeostasis, oxidative stress, and extensive lipid peroxidation [45]. To mitigate this cellular toxicity, the plant orchestrates a robust, tissue-specific antioxidant defense response. In roots, several *AarCAT* and *AarSOD* genes were broadly upregulated under both salt and saline-alkali treatments to combat soil-borne stress. In leaves, foliar tissues predominantly downregulated the broader gene families, relying instead on the specific, intense activation of genes like *AarCSD3*, *AarCAT3*, and *AarCSD4* to manage the foliar ROS load. Ultimately, in leaves, SOD, CAT, and POD enzyme activities increased with stress intensity, as did levels of non-enzymatic scavengers, including GSH, flavonoids, and phenolic acids [46,47,48]. Hierarchical clustering grouped antioxidant metabolites and enzyme activities with the high-stress T4 and T5 treatments, reduceding the plant growth and photosynthetic parameters. Mantel plot correlation tests identified *AarCAT3*, *AarCSD3*, *AarFSD3*, and *AarMSD3* as vital core genes associated with antioxidant response. Among these, *AarFSD3* and *AarMSD3* emerged as highly interlinked master regulators that showed strong correlations with GSH, CAT, SOD, and global ROS levels. These hub genes represent the core molecular machinery driving extreme salt tolerance in *A. argyi*, offering highly specific genetic targets for future molecular breeding programs aimed at engineering climate-resilient *A. argyi* cultivars [4,8,9]. This study provides the first genome-wide characterization of the CAT and SOD families in *A. argyi*. Thus, this study serves as a foundational genomic resource and hypothesis-generating framework, providing actionable breeding targets rather than a definitive mechanistic elucidation of salt tolerance in *A. argyi*.

## 4. Materials and Methods

### 4.1. Identification and Physicochemical Property Analysis of CAT/SOD Genes in A. argyi

In this study, the whole-genome data and annotation files of *A*. *thaliana* and *A. argyi* were downloaded from the TAIR database (https://www.arabidopsis.org/) and the *A. argyi* genome database (https://www.bic.ac.cn/IMP/, accessed on 4 April 2026), respectively. To comprehensively identify the members of the *A. argyi CAT/SOD* gene family, we performed a blast using the *Arabidopsis* CAT/SOD protein sequences as queries to identify the *CAT/SOD* gene family in *A. argyi* with structurally similar characteristics, using default parameters (e-value ≤  1 × 10^−5^). The conserved DNA-binding domains (PF00199, PF06628, PF00080, PF02777, and PF00081) were obtained from the protein family database, and TBtool 2.485 was used to perform the simple HMM search function to identify the conserved domains in *A. argyi* CAT/SOD protein sequences; the default parameter was a minimum hit e-value = 0.01.

### 4.2. Physicochemical Property Analysis of CAT/SOD Genes in A. argyi

The physicochemical properties were analyzed by using the ExPASy ProtParam tool (https://wcb.expasy.org/protparam, accessed on 4 April 2026) for each *CAT/SOD* candidate gene, including instability index, hydrophobicity, isoelectric point, and protein length [32]. Additionally, CELLO v2.5 was used to predict the subcellular localization of the proteins (https://cello.life.nctu.edu.tw/, accessed on 4 April 2026) by providing the *CAT/SOD* protein sequences.

### 4.3. Phylogenetic Analysis of CAT/SOD Genes in A. argyi

To perform the phylogenetic analysis, CAT/SOD protein sequences obtained from the model plant *A. thaliana* were obtained from the TAIR database. In addition, *CAT* and *SOD* protein sequences from *Artemisia annua*, *B. napus*, *B. rapa*, and *B. olerecea* were also obtained. Along with these sequences, CAT and SOD protein sequences from *A. argyi* were used for multiple sequence alignment through MEGA11 using the MUSCLE algorithm [40,49]. Phylogenetic analysis was performed by applying the neighbor-joining method and using the most appropriate substitution model with 1000 bootstrap replicates. The phylogenetic tree was visualized using iTOL (https://itol.embl.de/, accessed on 6 April 2026) [50].

### 4.4. Conservative Domain Analysis, Gene Structure and Motif Analysis of CAT/SOD Genes in A. argyi

The *A. argyi* genome annotation data in GFF format were used to perform gene structure analysis. The intron/exon structures of the 22 identified *CAT/SOD* genes were analyzed by TBtools. For further functional analysis of *AarCAT/SOD* proteins, the MEME suite (https://meme-suite.org/meme, accessed on 8 April 2026) was used; a maximum of 15 motifs were identified, with motif repetitions set to 0 or 1. TBtools was used to integrate and create a composite diagram of the gene structure and conserved domains using motif data from MEME. This diagram consists of the phylogenetic tree, conserved domains, motifs, and gene structure, providing a better overview of the functional implications and structural features of the *CAT/SOD* genes in *A. argyi*.

### 4.5. Chromosomal Localization, Gene Duplication and Collinearity Analysis of CAT/SOD Family Genes in A. argyi

The *A. argyi* genome was used to analyze gene density using TBtools, and the results are shown on the chromosome map. The positions of the *AarCAT/SOD* genes were analyzed using the GFF genome annotation file, and a distribution map of all *AarCAT and AarSOD* genes was generated. Collinearity analysis and gene duplication of the *AarCAT and AarSOD* family members were performed using the One-Step MCScanX method in TBtools. The Advanced Circos function was used to analyze the segmental duplication events and chromosomal distribution of the 24 *CAT/SOD* genes in *A. argyi*. Moreover, genome data and annotation files for *A. thaliana* and *A. annua* were used to examine the evolutionary relationships of segmental duplication events. The Simple Ka/Ks Calculator was used to calculate the Ka, Ks, and Ka/Ks values of the segmental duplication gene pairs. The following formula was used to calculate the estimated divergence time: T = Ks/2 λ × 10^−6^ Mya (where λ = 1.5 × 10^−8^) [51].

### 4.6. GO Enrichment Analysis, Promoter Analysis, and Protein Interaction Networks of CAT/SOD Genes in A. argyi

The 24 *CAT/SOD* protein sequences of *A. argyi* were used for Gene Ontology (GO) annotations using Blast2GO64 (https://www.blast2go.com/), classifying genes into cellular components, biological processes, and molecular functions (*p*  <  0.05). TBtools was used for data visualization [52]. The PlantCARE database was used to analyze the promoter regions (2000 bp upstream of the start codon) retrieved from the *A. argyi* genome. The STRING online tool was used for protein–protein interaction (PPI) networks [53].

### 4.7. Three-Dimensional Protein Structure of AarCATs/AarSODs

FASTA sequence of *AarCAT* and *AarSOD* proteins was submitted to SwissModel for structural modeling. Structural templates were identified using the Protein Data Bank (PDB). Model quality was analyzed using GMQE scores, QMEAN, and Ramachandran plots. Further refinement and molecular dynamics simulations were conducted using iMOD.

### 4.8. Transcriptome Analysis Based on RNA-Seq Data

The RNA-seq expression profiles of *AarCAT/SOD* genes in different plant tissues were mined from the transcriptome data. To reveal the expression profile of *CAT* and *SOD* genes in *A. argyi*, RNA-seq data were obtained from the NCBI BioProject database (PRJNA804653, PRJNA1135560, and PRJNA722539). Furthermore, data on salt and saline–alkali stress in roots and leaves with three biological replicates used in our study were deposited into PRJNA1054765. Raw read counts were normalized using FPKM (fragments per kilobase of transcript per million mapped reads). Using the Cufflinks tool (v2.2.1), FPKM values were calculated to estimate the levels of gene expression. Prior to normalization, raw reads were filtered for quality using Trimmomatic and mapped to the *A. argyi* reference genome using HISAT2 v2.2.1. Only uniquely mapped reads were retained for quantification. Heatmaps were constructed with row scaling using TBtools software v2.485 based on log2(FPKM values), and each dataset had three replicates.

### 4.9. RNA Extraction and RT-qPCR Analysis

Total RNA from different samples of *A. argyi* was extracted using the EASYspin Plus Plant RNA Kit (AidLab, Beijing, China) following the manufacturer’s instructions. One microgram (1 µg) of quantified RNA was used for cDNA synthesis with the EasyScript All-in-One First-Strand cDNA Synthesis SuperMix for qPCR (One-Step gDNA Removal) (Transgen, Beijing, China) according to the manufacturer’s protocol. RT-qPCR was conducted on an ABI QuantStudio 5 (Thermofisher Scientific, Waltham, MA, USA) using TransStart Top Green qPCR SuperMix (Transgen, Beijing, China) and followed a previously published protocol [54]. Each 20 µL reaction contained 10 µL 2× SuperMix, 1 µL cDNA template, 0.4 µL of each gene-specific primer (10 µM), 0.4 µL 50× Passive Reference Dye II, and 7.8 µL nuclease-free water. Cycling conditions were 94 °C for 30 s, followed by 45 cycles of 94 °C for 5 s and 60 °C for 30 s. Three biological and three technical replicates were included, with *A. argyi* Actin genes used as the internal reference. Relative expression was calculated using the 2^−∆∆Ct^ method [54]. All primers are listed in Table S2.

### 4.10. Plant Materials, Growth Conditions and Light Treatments

The *A. argyi* (Xiang Ai cultivar) plant material used in this study was sourced from Qichun County, Hubei Province, and kept at the College of Life Sciences, Northwest Agriculture and Forestry University, Yangling, China. The root buds of *A. argyi* were grown in vermicompost, and after germination, seedlings were placed in a plant growth chamber under a 16 h/8 h light/dark photoperiod cycle at 23~26 °C. After 1 month of seedling growth, seedlings of the same height were selected and separated into two groups, one for different light treatments (blue, red, and white) and the other for salt treatment (0 mM, 50 mM, 100 mM, 150 mM, and 200 mM NaCl). Samples were collected at six time intervals (0, 1, 3, 6, 12, and 24 h) for RT-qPCR, and plants were harvested after 1 month for other biochemical attributes. Light-treated samples were also harvested after 1 month. All collected samples were immediately frozen in liquid nitrogen and stored at −80 °C.

### 4.11. Leaf Growth Attributes, Scanning Electron Microscopy (SEM) and Photosynthetic Pigments

After cleaning, fresh leaves were weighed using an electric balance, oven-dried at 70 °C for 72 h for dry weight, and shoot length was measured with a scale. For SEM analysis, leaves were placed in 100% ethanol for dehydration twice. A gold–palladium layer was applied (Hitachi E-1010 High-Tech Corporation, Tokyo, Japan), 5 min, followed by analysis with a scanning electron microscope (Hitachi SU-8010 High-Tech Corporation, Tokyo, Japan) to observe stomatal conductance. Stomatal length and width were measured using FIJI v2.28.0 software. Chlorophyll a and b were quantified following Lichtenthaler [41]. Fresh 0.5 cm leaf segments were soaked in 5 mL of 80% acetone overnight at −10 °C. Absorbance was recorded at 645 and 663 nm using a spectrophotometer, and pigment content was calculated using the following formulas:

Chl. a = [12.7(A663) − 2.69(A645)] × V/1000 × W

Chl. b = [22.9(A645) − 4.69(A663)] × V/1000 × W

Carotenoid = [(A480) + 0.114(A663) − 0.638(A645)]/2500

### 4.12. Gas Exchange Parameters

Gas exchange attributes, including net photosynthetic rate (Pn), intercellular CO_2_ concentration (Ci), transpiration rate (Tr) and stomatal conductance (gs) were recorded during sunny days (12:00–13:00) on fully expanded leaves using an Infrared Gas Analyzer LI-6800 Portable Photosynthesis System; (LI-COR Biosciences, Lincoln, NE, USA) [7].

### 4.13. Total Soluble Protein and Total Soluble Sugar

Fresh 0.5 g leaf samples were homogenized in 80% ethanol. Total soluble protein was quantified using Bradford’s method [42], with absorbance measured at 595 nm. Total soluble and reducing sugars were assessed using Anthrone reagent and O-toluidine following [43], with absorbance measured at 540 nm.

### 4.14. Determination of Antioxidants and ROS

POD and SOD assays were performed according to [46]. Ascorbate peroxidase (APX) and CAT were performed according to [47,48], respectively. Malondialdehyde (MDA) was measured using the TCA-TBA method [45]. Hydrogen peroxide (H_2_O_2_) was determined spectrophotometrically, and NBT staining was performed as described by [7]. DAB staining for in situ H_2_O_2_ detection was performed according to [44].

### 4.15. Statistical Analysis

The least significant difference (LSD) test was used to compare the mean values of the treatments with a significance level of *p* < 0.05, performed in Statisticx 8.1 software. GraphPad Prism software (v9.0) was used to create all Figures, and the heatmap was created using the TB tool. For the multivariate analysis and PCA graph, R Studio (version 2026.01) was utilized to perform Mental correlation.

## 5. Conclusions

This study delivers a comprehensive genomic and physiological blueprint of the antioxidant defense architecture of *A. argyi*. By characterizing the 22 core members of the *CAT* and *SOD* gene families, we uncover a uniquely tailored evolutionary lineage defined by strict structural conservation and precise intracellular localization. The evidence confirms that survival under severe saline conditions relies on a highly orchestrated, tissue-specific response rather than a passive biochemical trait. *A. argyi* successfully navigates profound osmotic and oxidative impairment by pairing rapid transcriptional reprogramming with a drastic metabolic pivot toward critical osmoprotectants like proline. Most importantly, our multivariate network analyzes isolated *AarFSD3* and *AarMSD3* as the hyper-connected master regulators dictating this entire redox network. These hub genes transcend basic physiological markers; they represent the fundamental genetic machinery of extreme salt tolerance, offering elite, actionable targets for the precise molecular breeding of climate-resilient crops.

## Figures and Tables

**Table 1:** Physicochemical characteristics and subcellular localization of *AarCAT* and *AarSOD* proteins in *A. argyi*.

Gene Name	Chromosome	Start	End	Protein Length (A.A)	Instability Index	Aliphatic Index	Protein Molecular Weight (Da)	pI	GRAVY	No. of Intron/Exons	Subcellular Localization
*AarCAT1*	Chr09	33247444	33250760	492	35.16	70.33	56,728.91	6.77	−0.605	7:08	Peroxisomal
*AarCAT2*	Chr17	24800416	24803735	492	35.16	70.12	56,714.89	6.77	−0.605	7:08	Peroxisomal
*AarCAT3*	Chr02	164530385	164536078	466	42.21	72.36	53,856.72	6.58	−0.553	7:08	Peroxisomal
*AarCAT4*	Chr02	11046944	11051653	499	38.57	70.72	57,355.61	6.4	−0.556	8:09	Peroxisomal
*AarCAT5*	Chr09	33262868	33267063	534	37.20	74.68	61,488.78	7.75	−0.52	7:08	Peroxisomal
*AarCAT6*	Chr17	24811116	24817044	543	36.37	77.75	62,569.20	8.13	−0.474	7:08	Peroxisomal
*AarCAT7*	Chr02	12585289	12589843	488	34.55	70.72	55,912.03	6.6	−0.553	7:08	Peroxisomal
*AarCAT8*	Chr10	130283145	130287621	499	37.69	74.43	57,635.20	6.42	−0.513	7:08	Peroxisomal
*AarCSD1*	Chr16	38918364	38922609	230	14.37	90.39	23,166.05	5.95	0.145	7:08	Chloroplast
*AarCSD2*	Chr0	98510	102036	231	15.42	90.43	23,232.16	6.08	0.148	7:08	Chloroplast
*AarCSD3*	Chr16	111550949	111553879	153	19.16	81.50	15,540.27	5.72	−0.216	7:08	Cytoplasm
*AarCSD4*	Chr08	111786843	111793297	244	23.56	93.81	26,311.15	6.21	0.016	9:10	Cytoplasm
*AarCSD5*	Chr08	110794703	110794778	151	20.25	83.84	15,763.69	5.80	−0.142	6:07	Cytoplasm
*AarFSD1*	Chr09	167002719	167007099	308	40.38	77.27	35,670.64	8.95	−0.447	9:10	Chloroplast
*AarFSD2*	Chr17	124490680	124493366	311	5	83.44	35,477.82	4.76	−0.500	7:08	Cytoplasm/Chloroplast
*AarFSD3*	Chr09	126324663	126324708	311	42.04	83.44	35,548.00	4.89	−0.507	7:08	Chloroplast/Cytoplasm
*AarFSD4*	Chr17	160756556	160758794	235	50.72	69.28	27,045.69	8.93	−0.519	5:06	Nucleus/Chloroplast
*AarFSD5*	Chr17	160780296	160782528	217	50.84	66.96	25,012.25	8.45	−0.565	9:10	Nucleus/Chloroplast
*AarMSD1*	Chr10	141041433	141044518	227	33.85	88.59	25,248.82	7.84	−0.289	5:06	Mitochondria
*AarMSD2*	Chr01	184103180	184106289	227	30.48	88.59	25,256.85	8.47	−0.288	5:06	Mitochondria
*AarMSD3*	Chr01	182533417	182536634	227	32.93	89.87	25,199.79	8.47	−0.283	5:06	Mitochondria
*AarMSD4*	Chr17	125732828	125736360	157	36.68	88.22	17,881.54	9.30	−0.333	5:06	Mitochondria

## Data Availability

All materials and data are available upon request. RNA-seq raw data (seven tissues and MeJA-treated leaves, 300 µmol/L) are deposited in the NCBI SRA and BioProject databases under accessions PRJNA804653, PRJNA1135560, PRJNA1054765, and PRJNA722539.

## Supplementary Materials

The following supporting information can be downloaded at: https://www.mdpi.com/article/10.3390/ijms27177748/s1.
